# Overexpression of Collagen Triple Helix Repeat Containing 1 (CTHRC1) is associated with tumour aggressiveness and poor prognosis in human non-small cell lung cancer

**DOI:** 10.18632/oncotarget.2421

**Published:** 2014-09-29

**Authors:** Zunfu Ke, Weiling He, Yuanhui Lai, Xuefeng Guo, Sharon Chen, Shuhua Li, Yuefeng Wang, Liantang Wang

**Affiliations:** ^1^ Department of Pathology, the First Affiliated Hospital of Sun Yat-sen University, Guangzhou, Province Guangdong, P.R. China; ^2^ Gastrointestinal Surgery, the First Affiliated Hospital of Sun Yat-sen University, Guangzhou, Province Guangdong, P.R. China; ^3^ Vascular and Thyroid Surgery, the First Affiliated Hospital of Sun Yat-sen University, Guangzhou, Province Guangdong, P.R. China; ^4^ Department of Gastrointestinal Surgery, the Sixth Affliated Hospital of Sun Yat-Sen University, Guangzhou, Guangdong; ^5^ Department of Molecular and Medical Pharmacology, University of California, Los Angeles, 570 Westwood Plaza, Los Angeles, CA USA

**Keywords:** CTHRC1, non-small cell lung cancer, β-catenin, metastasis, prognosis

## Abstract

Collagen triple helix repeat-containing 1 (CTHRC1), a novel oncogene, was identified to be aberrantly overexpressed in several malignant tumors. However, the expression profile of CTHRC1 and its clinical significance in non-small cell lung cancer (NSCLC) remain unknown. In this study, we showed that CTHRC1 was evidently overexpressed in human NSCLC tissues and NSCLC cell lines at the protein and mRNA level. Ectopic up-regulation of CTHRC1 in cancer cells resulted in elevated invasive and proliferative abilities, which were attenuated by the specific CTHRC1 siRNA. The biological effect of CTHRC1 on metastasis and proliferation was mediated by the activation of the Wnt/β-catenin pathway. Furthermore, CTHRC1 immunoreactivity was evidently overexpressed in paraffin-embedded NSCLC tissues (212/292, 72.60%) in comparison to corresponding adjacent non-cancerous tissues (6/66, 9.09%) (*p*<0.001). Clinicopathologic analysis showed that CTHRC1 expression was significantly correlated with differentiation degree (*p*<0.001), clinical stage (*p*<0.001), T classification (*p*<0.001), lymph node metastasis (*p*=0.013) and distant metastasis (*p*<0.001). Kaplan-Meier analysis revealed that patients with high CTHRC1 expression had poorer overall survival rates than those with low CTHRC1 expression. Multivariate analysis indicated that CTHRC1 expression was an independent prognostic factor for the overall survival of NSCLC patients. Collectively, CTHRC1 plays important roles in NSCLC progression, and the evaluation of CTHRC1 expression could serve as a potential marker for metastasis progression and prognosis in NSCLC patients.

## INTRODUCTION

Lung cancer is the leading cause of cancer-related mortality worldwide [1] and it is becoming a common malignancy in developing countries such as China because of air pollution and a high cigarette smoking rate [2]. According to pathological classification based on distinct aetiological and morphologic characteristics, lung cancer is divided into small cell lung cancer (SCLC) and non-small cell lung cancer (NSCLC). The latter accounts for 85% of all lung cancer patients [3]. Despite advances in developing more efficient surgical techniques and novel chemotherapeutic interventions, the long-term survival rate of NSCLC patients remains poor [4, 5]. The major cause of extremely poor prognosis in NSCLC patients is mostly attributed to its ability to metastasize to distant organs by complex molecular mechanisms [6]. Furthermore, two-thirds of NSCLC patients present locally advanced or metastatic stage at diagnosis, often leading to death within a few months after diagnosis [7]. Therefore, there is an urgent need to exploit novel molecular markers that can better predict metastasis and prognosis for NSCLC.

Metastasis is the main cause of cancer-related mortality, and it involves a series of steps including invasion and migration, intravasation, circulation in bloodstream, extravasation, colonization growth in specific organs and secondary tumor formation [8]. At the early stage of metastasis, tumor cell mobility and invasive ability relates to loss of intercellular adhesive properties, epithelial-to-mesenchymal transition (EMT), proteolytical disruption of the basement membrane and degradation of the extracellular matrix (ECM) [9]. During the early progression of metastasis, activation of the Wnt/β-catenin pathway may promote the migration and invasion of cancer cells by up-regulating matrix metalloproteinases MMP-7 and MMP-26 and down-regulating cell-cell contact protein E-cadherin [10–13]. However, the molecular mechanisms underlying the aberrant activation of Wnt/β-catenin pathway in NSCLC have not been elucidated.

Collagen triple helix repeat containing-1 (CTHRC1) was found to be ubiquitously expressed in numerous cell types such as fibroblasts and smooth muscle cells, and aberrantly up-regulated in several malignant tumors, including melanoma, and cancers of the gastrointestinal tract, breast, thyroid, liver and the pancreas [14–19]. Several reports have documented that cell surface anchored CTHRC1 can stabilize the physical interaction between Wnt ligands and Frizzled receptors, and selectively activate the non-canonical Wnt pathway to regulate cell motility and taxis [16, 20]. Forced expression of CTHRC1 may contribute to repairing the injured tissue by promoting cell migration and inhibiting the collagen matrix synthesis [21]. In human beings, CTHRC1 shares 92% of homolog sequences compared to that of the rat [17]. Tang et al [17] reported that CTHRC1 expression was significantly higher in primary invasive and metastatic melanomas but not in benign nevi or non-invasive specimens, and the knockdown of CTHRC1 resulted in a decrease in migration ability in melanoma cancer cells in vitro. Recently, Kharaishvili et al [22] demonstrated that CTHRC1 up-regulation was closely correlated with breast cancer carcinogenesis and bone metastasis. However, currently, little information has addressed the clinical significance of CTHRC1 and the molecular mechanisms by which CTHRC1 promotes the invasion and metastasis of NSCLC.

In this study, we found that CTHRC1 expression was significantly increased in NSCLC cells and surgical tissues, and was closely associated with tumor metastasis. Moreover, knock-down and ectopic expression of CTHRC1 showed that CTHRC1 promoted the invasion and migration of NSCLC cells in vitro via the Wnt/β-catenin signaling pathway, and this is further verified by the correlation analysis between CTHRC1 expression and both lymph node and distant metastasis in archived patient samples. Multivariate analysis demonstrated that overexpression of CTHRC1 was an independent prognostic factor for NSCLC patients.

## RESULTS

### Elevated expression of CTHRC1 in NSCLC

**Figure 1:**
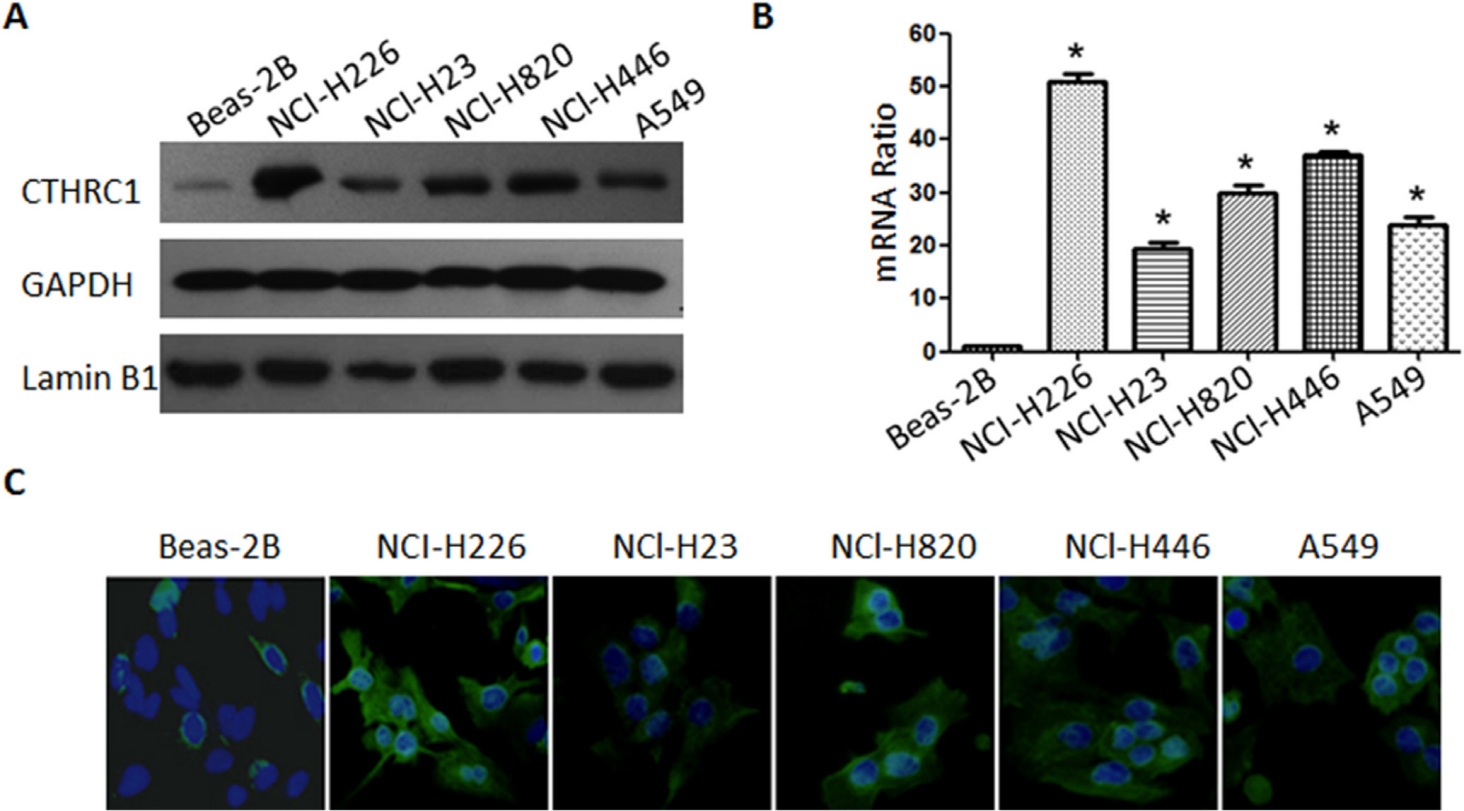
Protein and mRNA expression characteristics of CTHRC1 in BEAS-2B and NSCLC cell lines **(A)** CTHRC1 protein was detected by Western blot in BEAS-2B and five NSCLC cell lines (NCI-H226, NCl-H23, NCl-H820, NCl-H820 and A549). The GAPDH and Lamin B1 genes were used as internal controls for cytosolic fraction and nuclear fraction, respectively. **(B)** Real-time PCR analysis of CTHRC1 mRNA levels in BEAS-2B and NSCLC cell lines. Expression intensity of CTHRC1 was normalized for β-actin. Error bars represent mean±SD from three independent experiments (*p<0.05). **(C)** Immunofluorescent staining showed that the CTHRC1-positive signal was present in the cytoplasm of BEAS-2B and NSCLC cell lines (Original magnification, ×400).

**Figure 2:**
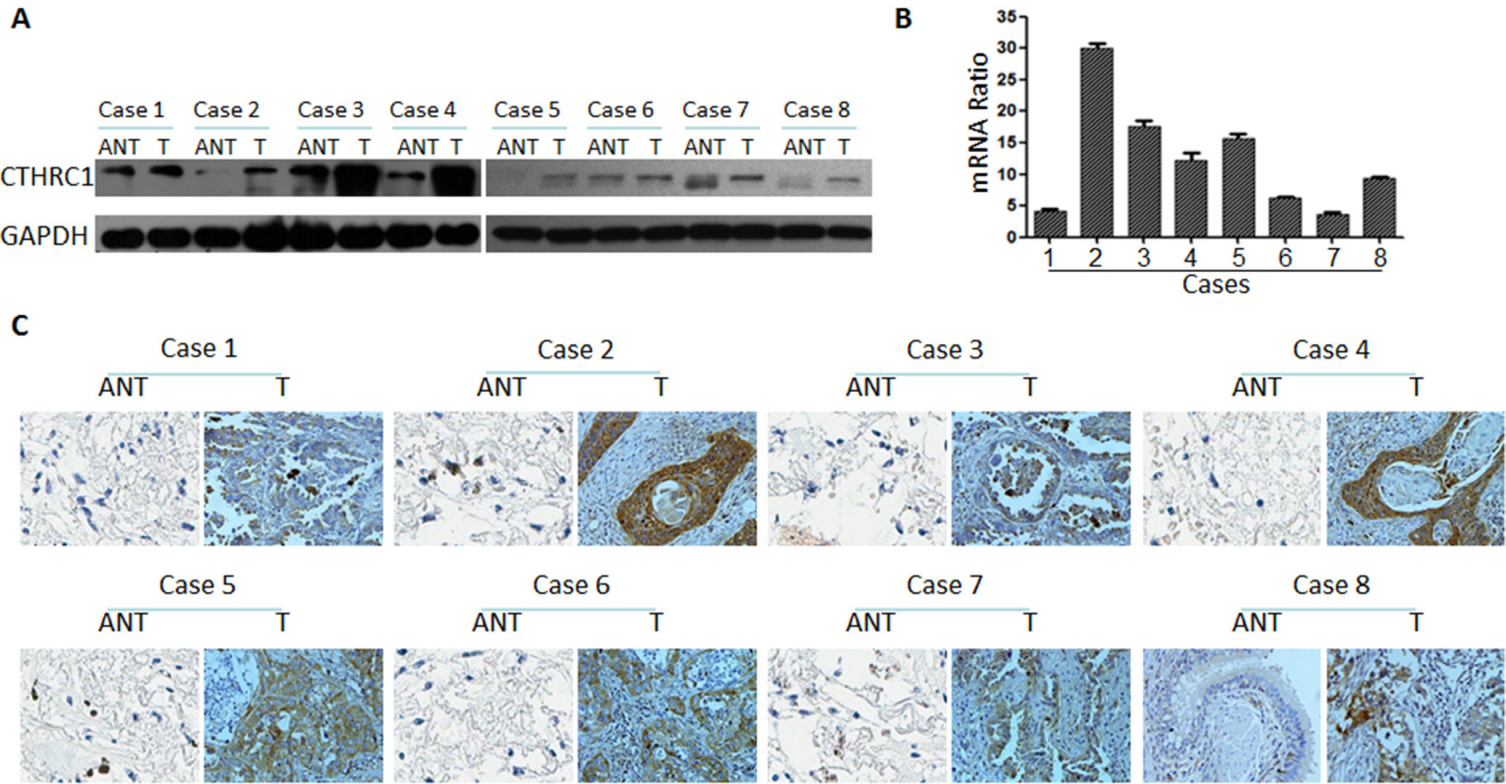
CTHRC1 overexpression in primary NSCLC tissues compared to the corresponding adjacent non-cancerous tissues from the same patient **(A)** Western blotting analysis of CTHRC1 expression in each of the primary NSCLC tissue (T) and adjacent non-cancerous tissue (ANT) from the same patient. **(B)** The average tumor: adjacent non-cancerous tissue (T:ANT) ratios of CTHRC1 expression were quantified by real-time RT-PCR. Expression levels were normalized for β-actin. Error bars represent mean ± SD from three independent experiments (*p<0.05). **(C)** IHC confirmed that the CTHRC1 protein was significantly elevated in primary NSCLC tissues (T) compared to that of corresponding adjacent non-cancerous tissues (ANT) from the same patient.

To investigate CTHRC1 expression traits in lung cancer, we comparatively analyzed the CTHRC1 protein and mRNA profiles in different lung cancer cell lines and samples. As shown in Figure 1A and B, Western blot and real-time RT-PCR analysis revealed that all lung cancer cell lines, including NCI-H226, NCl-H23, NCl-H820, NCl-H446 and A549, exhibited higher levels of CTHRC1 expression compared to that of primary human normal lung epithelial cells (Beas-2Bs) at both the protein and mRNA levels. Furthermore, the fluorescent intensity of CTHRC1 in the five NSCLC cell lines studied was significantly stronger than that of BEAS-2Bs (Fig. 1C). Particularly, its expression was most pronounced in strongly metastatic cell lines (NCI-H226, NCl-H820 and NCl-H446) compared to that of non- or low-metastatic lung cancer cell lines (NCl-H23 and A549) (Fig. 1B). Further comparative analysis demonstrated that CTHRC1 was differentially up-regulated in all eight detected samples paired with corresponding non-cancerous tissues from the same patient (Fig. 2A and B), and this was further confirmed by the immunohistochemical results (Fig. 2C). With these findings, our results indicate that CTHRC1 is up-regulated in NSCLC.

### CTHRC1 promotes migration and invasion of NSCLC cells in vitro

**Figure 3:**
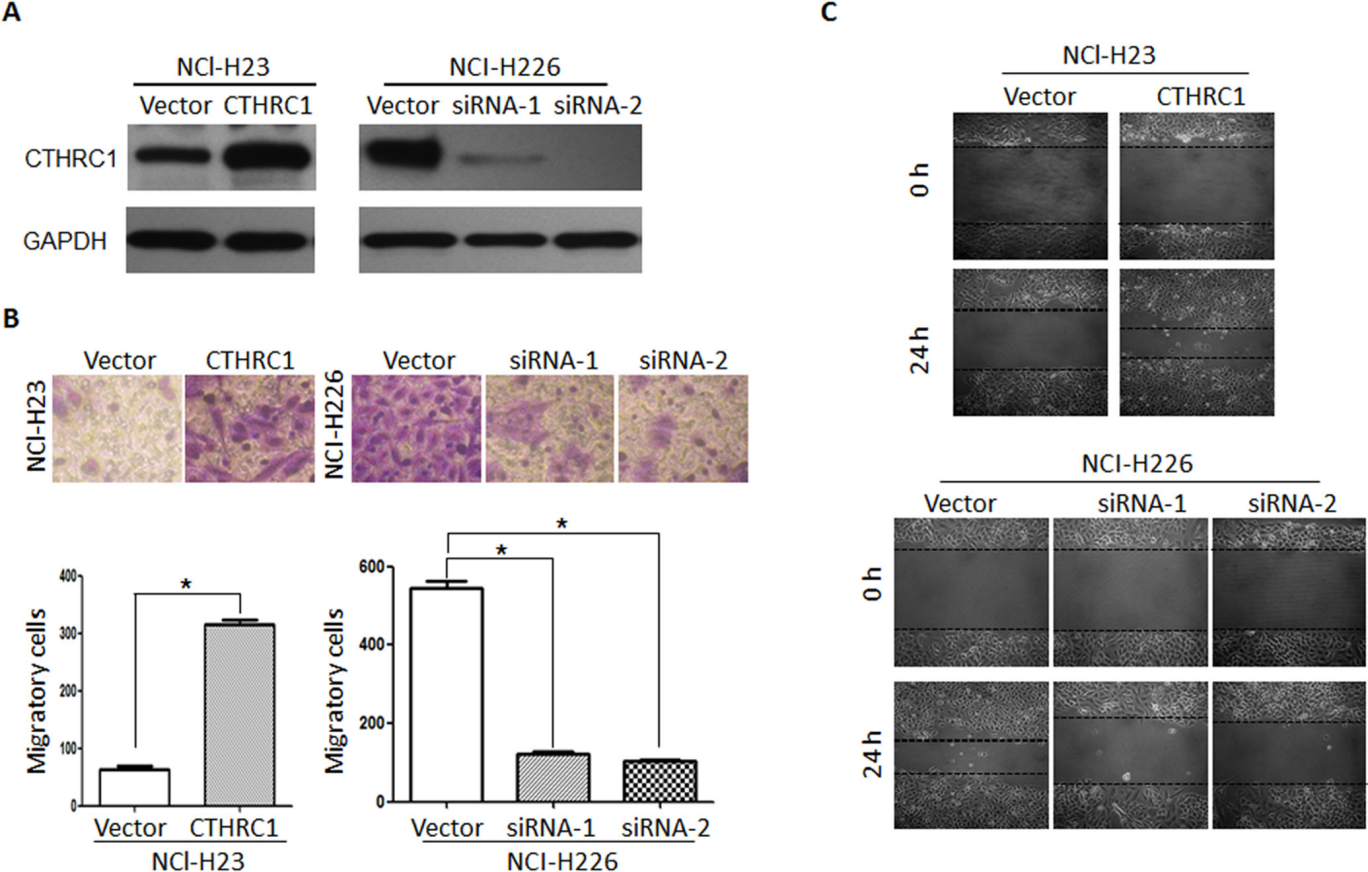
Aberrant expression of CTHRC1 promotes the invasion and migration of NSCLC cells in vitro **(A)** NCl-H23 and NCl-H226 cells were transfected with pcDNA3.1-CTHRC1 and CTHRC1-siRNA, respectively. CTHRC1 protein expression was analyzed by using the Western blot method. GAPDH was employed as an inner control. **(B)** The effect of CTHRC1 expression changes on the invasive ability in indicated cells was detected by Matrigel invasion assay. (Upper) Cells that adhered to the lower surface of the filtered were stained with hematoxylin. (Down) The number of migrated cells was from three independent experiments. **(C)** The migratory speed of CTHRC1-expressing NCl-H23 cells and CTHRC1-siRNA-expressing NCl-H226 cells was monitored through a scratch wound assay at different times. The representative images are from three independent experiments.

To investigate the biological effect of CTHRC1 deregulation on the invasiveness of lung cancer cells, in vitro gain-of-function or loss-of-function analyses were performed using migration and invasion assays. The efficacy of pcDNA3.1-CTHRC1 and CTHRC1-siRNA on CTHRC1 was confirmed by Western blot analysis (Fig. 3A). As shown in Figure 3B, CTHRC1 overexpression drastically strengthened the invasive ability of NCl-H23 cells compared to that of the vector control. Wound healing assay also showed that CTHRC1 overexpression significantly increased the migratory speed of NCl-H23 cells compared to that of the vector control (Fig. 3C). In contrast, the silencing of CTHRC1 expression weakened the invasive capability of NCI-H226 cells with a high metastatic phenotype (Fig. 3B and C). Our data indicate that CTHRC1 contributes greatly to the development of lung cancer metastasis and invasion.

### CTHRC1 increases proliferation in lung cancer cells

**Figure 4:**
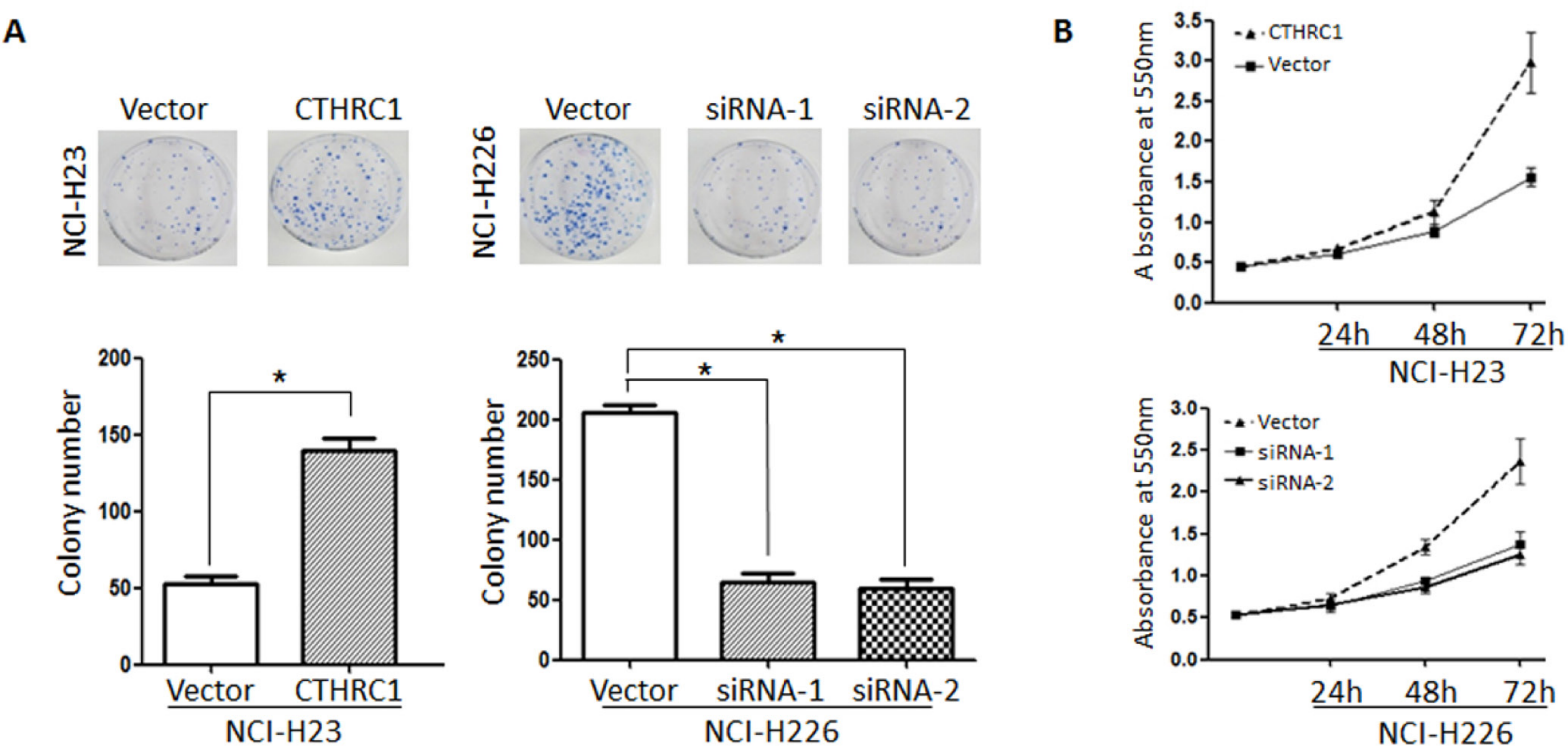
Effect on the soft agar colony formation and proliferation ability of NSCLC cells following CTHRC1 ectopic expression **(A)** Colony forming ability of NCl-H23 cells transfected with pcDNA3.1-CTHRC1 and NCl-H226 transfected with CTHRC1-siRNA. Data are mean ± SD of three independent transfections. **(B)** Effect of CTHRC1 on NSCLC cell growth was evaluated by MTT assay. The line charts showed the relative MTT absorbance, which indicated the cellular viability.

In order to examine the role of CTHRC1 in NSCLC cell proliferation, we evaluated the effect of CTHRC1 on NSCLC cell clonogenic assay and cell growth. As shown in Figure 4, up-regulation of CTHRC1 significantly increased colony formation in NCI-H23 cells by 263.46% compared to that of the control group. Furthermore, cell viability was also enhanced after transfection with pcDNA3.1-CTHRC1. On the contrary, down-regulation of CTHRC1 led to the drastic decline in colony formation and cell viability in NCI-H226 cells.

### CTHRC1 mediates NSCLC aggressiveness via GSK-3β/ β-catenin pathway

**Figure 5:**
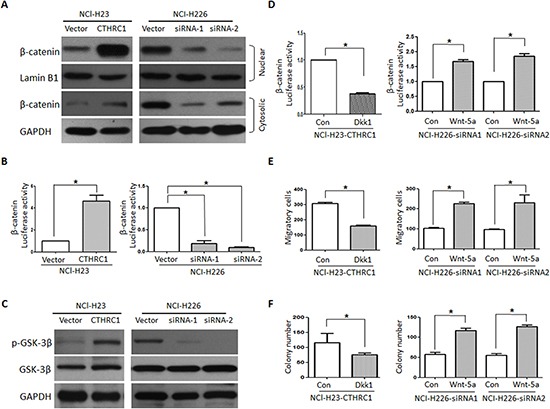
CTHRC1 promotes aggressiveness of NSCLC via activating Wnt/β-catenin pathway **(A)** CTHRC1 induces β-catenin nuclear translocation. The subcellular localization of β-catenin was observed through Western blot. **(B)** CTHRC1 enhances β-catenin/TCF transcriptional activity, which was evaluated by the TCF-responsive promoter reporter (TOP-flash) or nonresponsive control reporter (FOP-flash); The luciferase activity was measured as the ratio of TOP/ FOP. Relative luciferase activity is presented as the mean ± SD from each sample after normalizing to the control. The asterisk indicates statistical significance (*p*<0.01). **(C)** The Ser-9 phosphorylation level of GSK-3β was modulated by CTHRC1 in NSCLC cells. **(D), (E) and (F)**. Dkk1 (Wnt/β-catenin inhibitor) decreased CTHRC1-mediated β-catenin/TCF transcriptional activity **(D)**, migration **(E)** and proliferation ability **(F)**. On the contrary, Wnt-5a increased CTHRC1-siRNA-mediated β-catenin/TCF transcriptional activity **(D)**, migration **(E)** and proliferation ability **(F)**.

Based on the vital role of the Wnt/β-catenin pathway in metastasis, we then explored whether CTHRC1 activates Wnt/β-catenin signaling and if the Wnt/β-catenin pathway is involved in CTHRC1-mediated metastasis. In the canonical Wnt/β-catenin pathway, the hallmark of Wnt signaling activation is β-catenin's nuclear translocation, where it forms a complex with a specific T-cell factor/lymphoid enhancer factor (Tcf/Lef) [23]. After up-regulating CTHRC1 expression in NCl-H23 cells with pcDNA3.1-CTHRC1, we observed the substantial accumulation of β-catenin in nucleus, suggesting that CTHRC1 might contribute to the activation of Wnt signaling (Fig. 5A). As expected, luciferase assays also demonstrated that CTHRC1 overexpression significantly increased the transcriptional activity of β-catenin/TCF in NCl-H23 cells, as determined by the β-catenin reporter system (TOP/FOP) (Fig. 5B). In contrast, the transfection of CTHRC1-siRNA reduced the β-catenin/TCF transcriptional activity in NCl-H226 cells (Fig. 5B). Also, Western blot analysis revealed that CTHRC1 could promote the phosphorylation of GSK-3β at Ser9 (Fig. 5C). Furthermore, the transcriptional activity of β-catenin/TCF substantially decreased in CTHRC1-expressing cells treated with Wnt/β-catenin inhibitor DKK1 and significantly increased in CTHRC1-siRNA- NCI-H226 cells treated with Wnt 5a (Fig. 5D). Also, the number of invasive cells and colonies dramatically decreased when CTHRC1-expressing cells were separately treated with DKK1 (Fig. 5E)

### Association of CTHRC1 overexpression with NSCLC's clinical aggressiveness

**Figure 6:**
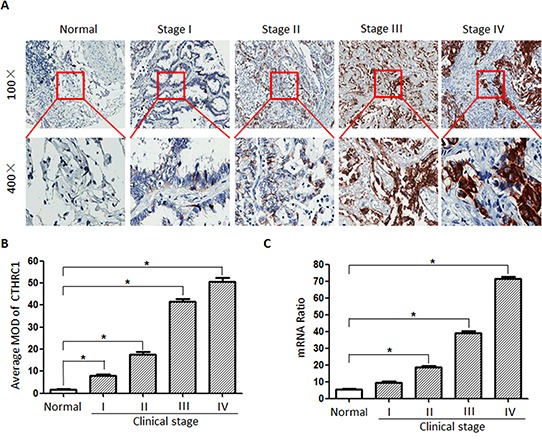
CTHRC1 protein and mRNA overexpression characteristics in archived paraffin-embedded NSCLC tissues from different clinical stages **(A)** CTHRC1 staining intensity gradually increased in accordance with the clinical stages from I to IV by IHC. Representative images from IHC analyses of CTHRC1 expression in normal lung tissues and primary NSCLC tissues. **(B)** Statistical analyses for the average MOD of CTHRC1 staining between normal lung tissues (72 cases) and NSCLC tissues of different clinical stages (*p<0.05). **(C)** CTHRC1 mRNA expression levels between normal lung tissues (72 cases) and NSCLC tissues of different clinical stages (*p<0.05).

To further elaborate the clinical significance of the above findings in NSCLC, 292 paraffin-embedded NSCLC tissue specimens were selected for CTHRC1 IHC staining. An overview of CTHRC1 expression and clinicopathological parameters is given in Table 1. CTHRC1 expression was significantly up-regulated in primary NSCLC tissues compared to their corresponding adjacent non-cancerous tissues. Semi-quantitative IHC analysis indicated that the MOD values of CTHRC1 staining in primary NSCLC were higher than that of normal lung tissues. CTHRC1 staining intensity gradually increased in accordance with the clinical stages from I to IV (*p*<0.001, Fig. 6A and B). And this was further confirmed by the Real-time PCR results (Fig. 6C). Furthermore, statistical analysis exhibited that high CTHRC1 expression was strongly correlated with the differentiation degree (*p*<0.001), clinical stage (*p*<0.001), T classification (*p*<0.001), lymph node metastasis (*p*=0.013) and distant metastasis (*p*<0.001) of patients with NSCLC, and this was further verified by the Spearman correlation analysis (Table 2). In contrast, there were no significant correlations between CTHRC1 high expression and age or gender. These data support the hypothesis that CTHRC1 is involved in the regulation of NSCLC's invasive ability.

**Table 1:** Correlation between CTHRC1 expression and clinicopathologic characteristics of NSCLC

Characteristics	CTHRC1 expression		Chi-square test
	High expression	Low expression	*p*-value
Normal lung tissues	6	66	<0.001
NSCLC tissues	212	80	
**Age(years)**			0.584
≤ 50	68	23	
> 50	144	57	
**Gender**			0.282
Male	151	62	
Female	61	18	
**Smoking**			<0.001
Yes	168	21	
No	44	59	
**Clinical stage**			<0.001
I	20	33	
II	102	41	
III	52	5	
IV	38	1	
**T Classification**			<0.001
T1	62	45	
T2	74	21	
T3	57	12	
T4	19	2	
**N Classification**			0.097
N0	106	53	
N1	78	20	
N2	15	4	
N3	13	3	
**Lymph node metastasis**			0.013
N0	106	53	
N1-3	106	27	
**M Classification**			<0.001
M0	174	79	
M1	38	1	
**EGFR mutation**			0.562
Positive	29	17	
Negative	35	16	
**K-ras mutation**			0.711
Positive	9	5	
Negative	49	34	

**Table 2:** Spearman correlation analysis between CTHRC1 and clinicopathologic parameters

	CTHRC1 expression level	
	Correlation coefficient	*p*-value
Differentiation degree	0.397	<0.001
Clinical stage	0.262	<0.001
T Classification	0.254	<0.001
N Classification	0.141	0.016
M Classification	0.219	<0.001
Lymph node metastasis	0.146	0.013

### CTHRC1 expression is closely correlated with poor overall survival time

**Figure 7:**
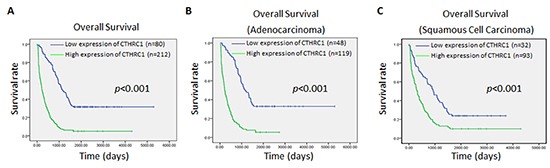
Kaplan-Meier survival analyses for NSCLC patients with low CTHRC1 expression versus high CTHRC1 expression **(A)** The cumulative overall survival differences between patients with high and low CTHRC1 expression. **(B)** and **(C)** Statistical significance of the difference between curves of CTHRC1 high-expressing and low-expressing patients was compared in adenocarcinoma **(B)** and squamous cell carcinoma **(C)** patient subgroups. P values were calculated according to the log-rank test.

To investigate the prognostic significance of high CTHRC1 expression in patients with NSCLC, the Kaplan-Meier method was used to evaluate the correlation between high CTHRC1 expression and the survival curve. Both the Kaplan-Meier method and the log-rank test revealed a significantly inverse correlation between high CTHRC1 expression level and patient survival (*p*<0.001), clearly disclosing that higher levels of CTHRC1 expression were associated with shorter survival rate (Fig. 7A). In both the squamous cell carcinoma group and adenocarcinoma group, patients with high CTHRC1 expression showed a shorter survival time in comparison to those with low CTHRC1 expression (*p*<0.001, Fig. 7B and C). Also, univariate and multivariate Cox regression analysis demonstrated that CTHRC1 expression level was an independent prognostic marker for NSCLC (Table 3). Thus, present data suggest that CTHRC1 may be a valuable biomarker for predicting the prognosis of NSCLC patients.

**Table 3:** Univariate and multivariate statistical analyses for various prognostic parameters in patients with NSCLC

Characteristics	No	Univariate analysis		Multivariate analysis		
		*p*-value	*Regression coefficient (SE)*	*p*-value	Relative risk	95% CI
**Expression of CTHRC1**		<0.001	0.378 (0.126)	<0.001	1.713	1.003–3.217
Low expression	80					
High expression	212					
**Differentiation degree**		<0.001	0.367 (0.121)	0.017	1.618	1.121–2.329
Well	79					
Moderately	141					
Poorly	72					
**Clinical stage**		<0.001	0.394 (0.132)	<0.001	1.517	1.127–1.798
I	93					
II	103					
III	57					
IV	39					
**T Classification**		<0.001	0.467 (0.223)	0.008	1.61	1.097–2.274
T1	107					
T2	95					
T3	69					
T4	21					
**N Classification**		<0.001	0.402 (0.195)	0.003	1.609	1.105–2.319
N0	159					
N1	98					
N2	19					
N3	16					

## DISCUSSION

Despite the recent advancement in therapeutic options, finding an accurate and efficient treatment option to monitor the progression of lung cancer still remains a challenge. One reason is the complex molecular mechanisms involved in the carcinogenesis and progression of lung cancer [24]. Therefore, elucidation of the exact molecular pathway involved in the pathogenesis of lung cancer will greatly contribute towards improving the outcome for patients. CTHRC1 is a secreted glycosylated protein that contains a NH2-terminal signaling peptide for extracellular secretion, a short collagen triple helix repeat of 36 amino acids, and a COOH-terminal globular domain [21]. Aberrant overexpression of CTHRC1 has been observed in several kinds of malignant tumors including breast cancer, gastric cancer and melanoma [17]. In the present study, our data demonstrated that CTHRC1 expression was up-regulated in human NSCLC tissues and cultured NSCLC cells compared to that of corresponding adjacent non-cancerous tissues and normal lung epithelial cells. Furthermore, high CTHRC1 expression was strongly correlated with the clinical stages, lymph node metastasis and distant metastasis. We also found that the CTHRC1 protein expression level in NCI-H226 cells with high metastatic ability was higher than that in NCl-H23 cells with low metastatic ability, and these results were further confirmed by real-time PCR and immunofluorescence assays. Multivariate analysis suggested that CTHRC1 may be an independent biomarker that can be used for NSCLC prognosis. Collectively, our results indicate that CTHRC1 is closely associated with tumor aggressiveness and may represent an independent prognostic biomarker for NSCLC patients.

Collected evidence supports the idea that CTHRC1 is closely linked with tumor invasion as well as metastasis [17, 25–28]. Using immunohistochemistry staining, Tang and Dai et al [17] found that CTHRC1 expression is absent in the benign nevi or noninvasive stages of melanoma (melanoma in situ) and dramatically increases in the invasive and metastatic melanoma. They demonstrated that the migratory behaviors of KZ-28 melanoma cells are inhibited by the specific CTHRC1 siRNA oligos. In addition, up-regulation of CTHRC1 resulted in an elevated invasion of colon cancer cells, but this process is significantly reduced by siRNA-mediated CTHRC1 knockdown [25]. Recently, Ma et al [26] further elucidated the role of CTHRC1 as a vital regulator of invasion and metastasis in the tumor microenvironment and documented that CTHRC1 overexpression greatly increases the likelihood of the aggressive feature in GIST patient. These studies suggest that CTHRC1 plays vital roles in the metastasis of malignant tumors; however, how it induces these phenotypes has remained unclear.

To further elucidate the biological roles of CTHRC1 as a putative oncogene, we examined the effect of CTHRC1 on cell proliferation and invasiveness in NSCLC cells using both gain of function and loss of function approaches. Since CTHRC1 participates in tissue remodeling in rheumatoid arthritis and injured arteries [21], we speculated that CTHRC1 may have a similar function in promoting NSCLC cell proliferation. In this study, up-regulation of CTHRC1 strongly enhanced the proliferation ability of NSCLC cells. Conversely, knockdown of endogenous CTHRC1 significantly reduced cell proliferation properties of NSCLC cells. Using cell migration assay, Pyagay et al demonstrated that elevated CTHRC1 levels enhance the migratory ability of fibroblasts and smooth muscle cells [21]. Turashvili et al showed that CTHRC1 was overexpressed in invasive lobular breast carcinoma compared to normal ductal and lobular cells [18]. In the present study, we have found that deregulation of CTHRC1 drove cell migration and promoted NSCLC cells to spread. Furthermore, CTHRC1's effect on promoting the aggressiveness of NSCLC cells was verified by IHC results on the significant correlations between CTHRC1 expression and lymph node metastasis or distant metastasis in NSCLC patients. Based on the above findings, CTHRC1 was thought to be an invasion-promoting protein and eventually contributed to NSCLC pathogenesis and metastasis. However, further exploration is needed to explain how CTHRC1 regulates cancer migration and invasiveness.

Next, to further elucidate the molecular mechanism of how CTHRC1 mediates the migration of NSCLC cells, we analyzed its effects on the Wnt/β-catenin signaling cascade. Tamamoto et al demonstrated that CTHRC1 activates the non-canonical Wnt/PCP signaling by forming complexes with the Wnt-Fzd and also inhibits the canonical pathway in mouse cochlear sensory hair cells [20]. To our knowledge, the binding of Wnts to Frizzled (Fzd) receptors plays an important role in many Wnt-mediated events [29]. Moreover, in most settings, the canonical Wnt/β-catenin signaling pathway has been extensively implicated as the regulator of tumor cell invasion and metastasis [30–32]. β-catenin is a main downstream effector of the canonical Wnt pathway. And it is recruited to the phosphorylation/destruction complex in the absence of Wnt ligands [33]. Overexpression of CTHRC1 led to a significant increase in Wnt/β-catenin transcriptional activity and an accumulation of nuclear β-catenin. Moreover, we found that treatment with DKK1, a specific inhibitor of Wnt/β-catenin signaling, diminished the migration ability and activity of Wnt/ β-catenin signaling in NSCLC cells induced by CTHRC1 overexpression. In addition, Wnt-5a could reverse CTHRC1-siRNA's effect on Wnt/β-catenin activity in NSCLC cells. These results suggest that CTHRC1-induced activation of Wnt/β-catenin signaling may account for its effect on cell proliferation and motility. GSK-3β, a multifunctional kinase of cancer, is constitutively activated because of tyrosine-216 phosphorylation, resulting in the phosphorylation of β-catenin [33]. Conversely, the phosphorylation of serine-9 deactivates GSK3β, inhibiting the proteasomal degradation of β-catenin [34–37]. Our preliminary studies also revealed that Ser-9 phosphorylation of GSK-3β was involved in the stability and transcriptional activity of CTHRC1-mediated β-catenin. On the basis of these results, we speculate that CTHRC1 promotes cell proliferation and migration via activation of the Wnt/β-catenin pathway. However, the detailed molecular mechanism regarding how CTHRC1 activates the Wnt/β-catenin pathway needs to be further explored.

In summary, we found that CTHRC1 may serve as a novel biomarker for NSCLC patients. The molecular mechanisms governing the role of CTHRC1 in NSCLC cell aggressiveness is mediated through the regulation of the Wnt/β-catenin pathway. Finally, CTHRC1 could be established as a potential target in NSCLC treatment, because its overexpression induces NSCLC aggressiveness.

## MATERIALS AND METHODS

### Cell lines

Primary normal lung epithelial cells (BEAS-2B) were purchased from American Type Culture Collection (ATCC) and cultured in a keratinocyte serum-free medium (Invitrogen, Carlsbad, CA) supplemented with epidermal growth factor (EGF) (Invitrogen), bovine pituitary extract, and antibiotics (100 μg/mL streptomycin and 100 U/mL penicillin). Lung cancer cell lines, including NCI-H226, NCl-H23, NCl-H820, NCl-H446, and A549, were maintained in Dulbecco's modified Eagle's medium (DMEM; Invitrogen, USA) supplemented with 10% fetal bovine serum (HyClone, Logan, UT). CTHRC1 overexpression plasmid pcDNA3.1-CTHRC1 and CTHRC1 siRNA (RiboBio, China) were transiently transfected using Lipofectamine 2000 (Invitrogen, USA).

### Tissue specimen selection

A total of 292“lung cancer” cases from 2000 to 2011 were chosen from the surgical pathology archives of the Affiliated First Hospital, Sun Yat-sen University. The age of these selected patients at the time of surgery ranged from 31 to 83 years (mean, 58.5 years). A follow-up was conducted by using hospital medical records and a telephone interview. According to the criteria of the World Health Organization (2004), the 292 cases were divided into 125 squamous cell carcinomas and 167 adenocarcinomas. Among the 292 cases with NSCLC, 72 cases contained primary carcinoma and corresponding normal lung tissue (Table 4). Another 97 NSCLC cases with EGFR (exons 18, 19, 20 and 21) and K-ras (condons 12 and 13) mutation information were obtained from November 2009 to May 2011. These 97 NSCLC cases were not detected to include T790M mutation in exon 20 before receiving radiotherapy or chemotherapy. Furthermore, eight pairs of fresh primary NSCLC tissues and adjacent non-cancerous lung tissues obtained from patients were used for real-time RT-PCR and Western blot. The medical ethics committee of Sun Yat-sen University approved the present retrieval method of cancer specimens.

**Table 4:** Clinicopathologic characteristics of patients with NSCLC

	All cases (%)		All cases (%)
**Age(years)**		**Gender**	
≤ 50	91 (31.16)	Male	213 (72.95)
> 50	201 (68.84)	Female	79 (27.06)
**Differentiation degree**		**Clinical stage**	
Well	79 (27.06)	I	93 (31.85)
Moderately	141 (48.29)	II	103 (35.27)
Poorly	72 (24.66)	III	57 (19.52)
		IV	39 (13.36)
**T Classification**		**N Classification**	
T1	107 (36.64)	N0	159 (54.45)
T2	95 (32.53)	N1	98 (33.56)
T3	69 (23.63)	N2	19 (6.51)
T4	21 (7.19)	N3	16 (5.48)
**Distant metastasis**		**Smoking**	
M0	253 (86.64)	Yes	189 (64.73)
M1	39 (13.36)	No	103 (35.27)
**Vital status (at follow-up)**		**Expression level of CTHRC1**	
Alive	37 (12.67)	Low expression	80 (27.40)
Death	255 (87.33)	High expression	212 (72.60)
**EGFR mutation**		**K-ras mutation**	
Positive	46 (47.42)	Positive	14 (14.43)
Negative	51 (52.58)	Negative	83 (85.57)

### Immunohistochemical staining and evaluation

Sections (4 μm) of formalin-fixed, paraffin-embedded tissues were made by using a rotary microtome (Leica, Wetzlar, Germany) and labeled with anti-CTHRC1 (rabbit polyclonal antibody, 1:1,000 dilution; Abcam (ab85739), USA). 3,3′-Diaminobenzidine tetrahydrochloride was added to visualize the staining reaction, followed by counterstaining with Mayer hematoxylin. The number of immunopositive cells was semiquantitatively estimated. First, staining intensity was scored: colorless (0), buff (1), brownish yellow (2), and dark brown (3). Second, the percentage of positive cells was scored: no positive cells (0), 10% positive cells or less (1), 11% to 50% positive cells (2), 51%to 75%positive cells (3), and more than 75% positive cells (4). The staining index was obtained by multiplying staining intensity score to the positive tumor cell score. The cutoff value for CTHRC1 was chosen based on a measure of heterogeneity using the log-rank test statistical analysis with respect to overall survival. An optimal cutoff value was identified: a staining index of four or greater was used to define tumors of high expression, and three or lower for low expression. A known positive control (human skin melanoma) and a negative control (an antibody dilution solution or an irrelevant rabbit IgG of the same isotype) were included with each run of staining to monitor batch consistency. The staining index was calculated using the Aperio ImageScope software (Aperio Technologies). To attain consistencies in IHC staining intensities, the mean optical density (MOD) was utilized to score the staining intensity of each slide. Five representative fields from each case were randomly selected to determine the MOD, representing the concentration of the stain or proportion of positive pixels within the whole tissue.

### Western blot and immunofluorescence

Tissue or cell lysates were prepared in radioimmunoprecipitation assay (RIPA) buffer. Immunoblot analyses were performed as earlier [23]. Blotted membranes were incubated with the antibodies for CTHRC1 (Abcam, Ltd., USA), β-catenin, p-GSK-3β (Ser-9), GSK-3β, GAPDH and LaminB1 (Abcam, Cambridge, UK) in 5% milk/TBST (tris-buffered saline Tween-20), and then with horseradish peroxidase (HRP)-conjugated secondary antibodies (Santa Cruz Biotechnology). Immunoreactive bands were observed by using a chemiluminescent substrate. For the immunofluorescence assay, cells cultured in the chamber slides were probed with CTHRC1. The fluorescein isothiocyanate (FITC)-conjugated anti-IgG was purchased from Molecular Probes. Cells were observed through an Olympus BX51 fluorescence microscope (Olympus, Tokyo, Japan).

### Total RNA extraction and Real-time RT-PCR

Total RNA was prepared from tissue specimens using the RNAeasy kit (Qiagen, USA). The amplification was carried out in a total volume of 20 μL containing LightCycler FastStart DNA Master SYBR green I (Roche, USA). Ct value (initial amplification cycle) of each standard dilution was plotted against standard cDNA copy numbers. By using the standard curves for each gene, the sample cDNA copy number was calculated according to the sample Ct value. Standard curves and PCR results were analyzed using ABI7000 software (Applied Biosystems, Foster City, CA, USA). Primers were CTHRC1: (sense) 5′-TGG ACA CCC AAC TAC AAG CA-3′ and (antisense) 5′-GAA CAA GTG CCA ACC CAG AT-3′. β-actin (primers: sense 5′-GCA TGG GTC AGA AGG ATT CCT-3′, antisense 5′- TCG TCC CAG TTG GTG ACG AT-3′) was used as an internal reference.

### Scratch wound assay

NSCLC cells transfected with vector control, CTHRC1 or CTHRC1 siRNA were plated in a 24-well plate and incubated under permissive conditions. After 90% confluence, the confluent cell monolayer was scratched using a 1-mL pipette tip to obtain a ‘wounded’ linear. Then, the cells were incubated in a serum-free medium for 48 hours. Cell migration was observed at different times and photos were taken under an inverted microscope.

### Transwell matrix penetration assay

The invasive ability was measured by using 24-well BioCoat cell culture inserts (Costar, New York, NY, USA) with 8-μm-porosity polyethylene terephthalate membranes coated with Matrigel Basement Membrane Matrix (Cultrex, MD, USA). 4×10^4^ lung cancer cells transfected with vector, CTHRC1 or CTHRC1 siRNA were then added to the upper chamber of the BioCoat cell culture inserts, and the medium containing serum (10%) was utilized as the chemoattractant in the lower chamber. At the end of the assay, the cells that did not migrate or invade the pores were removed with a cotton swab. After fixing with 1% paraformaldehyde and staining with hematoxylin, cells adhering to the lower membrane of the inserts were counted and observed under an IX71 inverted microscope (Olympus Corp,Tokyo, Japan). Cell counts are expressed as the average number of cells per field of view. Three independent experiments were performed and the data are presented as the average ± standard deviation.

### Colony formation assay

After 48 hours of transfection, lung cancer cells transfected with vector, pcDNA3.1-CTHRC1 or CTHRC1 siRNA were trypsinized, counted, and seeded in 60 mm dishes at 200 cells per dish. The medium was changed every four days. After two weeks of incubation under the conditions of 37°C and 5% CO2, colonies were fixed with acetic acid-methanol (1:4) and stained with crystal violet prior to being counted. Colonies containing more than 50 cells were counted under a microscope.

### Cell viability assay

Cell viability assays were performed according to the following method. In the 96-well plates, the logarithmically growing cells such as NCI-H23 and NCI-H226 (1×105/L) were seeded in each well at 150 μL/well 24 hours before transfection. 20 μL of filter-sterilized 3-(4,5-Dimethylthiazol-2-yl)-2, 5-diphenyltetrazolium bromide (MTT, 10 mg/mL) was dissolved in PBS and added to each well, and then the supernatant was gently aspirated after incubation for three hours at 37°C. Eventually, 200 μL of DMSO (dimethyl sulfoxide) was added to each well and oscillated for ten minutes to dissolve formazan crystals. Absorbance of OD from the plates was read at 550 nm through a UV spectrophotometer at 24, 48 and 72 hours after transfection. The assay was performed three times independently.

### Luciferase reporter gene assay

For the reporter gene assay, cells seeded in 24-well plates were transfected with the firefly luciferase reporter gene construct (TOP or FOP; 200ng), and 1ng of pRL-SV40 Renilla luciferase (as an internal control). Cell extracts were prepared 24 hours after transfection, and luciferase activity was measured using the Dual-Luciferase Reporter Assay System (Promega, USA).

### Coculture with Dkk1 and Wnt-5a

We cultured NCl-H23-CTHRC1 cells together with Dkk1 (R&D, USA) at the concentration of 15 ng/mL. Wnt-5a-conditioned medium (Wnt-5a-CM) was produced from L cells transfected with pGKWnt-5a. The medium was centrifuged at 1,000g for 15 minutes and filtered through a nitrocellulose membrane. Then, cells were treated with Wnt-5a CM for 24 hours and Wnt signaling was monitored by various assays, including Western blotting and luciferase reporter gene assays.

### Statistical analysis

Statistical analysis was undertaken with SPSS 16.0 software. The Pearson χ^2^ test was used to assess the correlation between CTHRC1 expression and clinicopathological parameters. Unpaired two-tailed Student's t-test was used to determine the statistical relevance between groups. Survival curves were plotted using the Kaplan-Meier method and compared with the log-rank test. Univariate and multivariate analyses were carried out by the Cox proportional hazards regression model. P values of less than 0.05 were considered statistically significant.
